# AAO-assisted synthesis of highly ordered, large-scale TiO_2_ nanowire arrays via sputtering and atomic layer deposition

**DOI:** 10.1186/s11671-015-0872-9

**Published:** 2015-04-08

**Authors:** Zhao Yao, Cong Wang, Yang Li, Nam-Young Kim

**Affiliations:** Department of Electronic Engineering, Kwangwoon University, 20 Gwangun-Ro, Nowon-gu, Seoul 139-701 Republic of Korea

**Keywords:** Titanium oxide, Nanowire arrays, Anodic aluminum oxide, Atomic layer deposition

## Abstract

Highly ordered nanoporous anodic aluminum oxide (AAO) thin films were fabricated in oxalic acid under a constant voltage via a two-step anodization process. To investigate the high-aspect-ratio (7.5:1) filling process, both sputtering and atomic layer deposition (ALD) were used to form TiO_2_ nanowires. Field emission scanning electron microscopy and high-resolution transmission electron microscopy images indicated that mushroom-like TiO_2_ structures were sputtered onto the AAO template surface, and the ALD-coated TiO_2_ exhibited fine filling results and clear crystal grain boundaries. Large-scale and free-standing TiO_2_ nanowire arrays were liberated by selectively removing the aluminum substrate and AAO template via a wet etching process with no collapsing or agglomeration after the drying process. ALD-deposited TiO_2_ nanowire arrays that were 67 nm in diameter and 400 nm high were transferred from the AAO template. The ALD process enabled the rapid, simple synthesis of highly ordered TiO_2_ nanowire arrays with desired parameters such as diameter, density, and thickness determined using diverse AAO templates.

## Background

One-dimensional TiO_2_ thin films with nanoscale structures, such as nanotubes, nanorods, and nanowires, present a variety of applications, including catalysis [1-3], gas sensing [4,5], and energy harvesting [6,7], due to their large surface-to-volume ratio, convenient band gap, and quantum confinement effects. Research into synthesizing TiO_2_ materials with nanoscale structures has significantly increased in recent years. Areas of focus include hydrothermal methods [8,9], pulsed laser deposition (PLD) [10], and the anodization of titanium films [11,12]. In addition, an easily controlled template-assisted growth method has also drawn significant interest because the template can be used to directly form a desired nanostructure. Using a template with a specified shape, diameter, density, and arrangement allows a large number of desired targets to be duplicated once the specific template structure has been formed. Even large-scale films can be easily prepared via a template synthesis. Various materials such as lithographed polymers [13], natural materials [14], and anodized metals (e.g., Al, Ti, and Mg), many of which are abundantly available, can be used for the template [11-16]. AAO thin films prepared via the two-step anodization of Al have a highly ordered honeycomb porous structure, and their preparation is more easily controlled than other materials. Compared to other strategies, this method is a simple, fast way to grow patterned nanostructure.

In AAO-assisted synthesis of nanostructures, sol–gel and electrochemical deposition are both well-known liquid-phase deposition methods for forming TiO_2_ nanowires [17,18]; however, both methods require drying and sintering processes to promote crystallinity. During the sintering process, the templates are fired, which complicates their removal. While AAO templates are removed using acid or alkaline solutions, the nanowire or nanotube arrays would most likely agglomerate, which may destroy their regular, vertical alignment [17]. In contrast, physical vapor deposition (PVD) processes can synthesize single-crystalline structures; however, the pattern size is usually limited by the template or photoresist aspect ratio due to self-limiting step coverage. An aspect ratio above 5:1 is generally not attainable using e-beam evaporation and sputtering system. Therefore, either the nanostructure size or height is limited by the PVD process. Consequently, vapor-phase deposition via chemical vapor deposition (CVD) or ALD exhibits potential for synthesizing nanowires or nanorods because it is not subject to such aspect ratio constraints. Combining ALD with AAO templates is the most promising strategy for synthesizing nanostructures. However, in previous researches [1,17,19], it shows difficulty to prevent the nanowires from agglomeration after a wet etching process. Otherwise, the height of the nanowires is limited to approximately 100 nm [13,20].

In this study, ALD was compared to PVD as a step coverage problem by filling 500-nm-thick nanoporous AAO thin films with 67-nm-diameter pores as templates for the TiO_2_ nanowires. The sputtering and ALD deposition rates were reduced to suit this high-aspect ratio. After the TiO_2_ deposition, the surface and cross-sectional structures of the TiO_2_ and Al_2_O_3_ mixtures were investigated via field emission scanning electron microscopy (FE-SEM) and elemental mapping, and the interface between the AAO and TiO_2_ were characterized via X-ray diffraction (XRD) and high-resolution transmission electron microscopy (HR-TEM). After the TiO_2_ nanowire arrays liberated from AAO templates and transferred onto Si substrate, highly ordered, large-scale, and vertically aligned nanostructures were achieved without agglomeration.

## Methods

**Figure 1:**
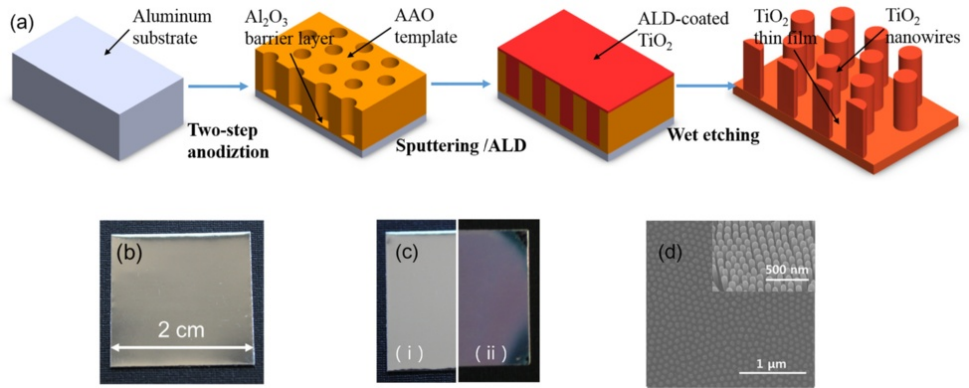
**Processes for fabricating highly ordered, large-scale TiO**_**2**_ **nanowire arrays. (a)** Schematic diagrams of the process to produce the nanowire arrays, **(b)** photograph of a 2 cm × 2 cm AAO template, **(c)** top view of the AAO templates after coating TiO_2_ via (i) sputtering and (ii) ALD with a mean pore diameter of 67 nm, and **(d)** top and cross-sectional SEM images of the highly uniform and free-standing TiO_2_ nanowire arrays.

The nanoporous AAO templates were fabricated via a prototypical two-step anodization electrochemical method using high-purity (99.999%) aluminum foil in a 0.3 M oxalic acid electrolyte at a temperature of 0.2°C under a constant anodization voltage of 40 V for 10 min. Next, a 500-nm-thick fine, self-ordered, hexagonal, nanoporous structure formed on the aluminum foil surface as a patterned template for nanorods, nanotubes, or nanowires. TiO_2_ thin films were deposited onto the nanoporous template via sputtering (SPS 4150, UL-TECH, Daegu, Korea) and ALD (TFS 200, BEN-EQ, Vantaa, Finland). For the sputtering process, the sputtering power, Ar flow rate, and chamber pressure were 2 kW, 80 sccm, and 4e − 6 Torr, respectively. During the ALD process, single-crystalline TiO_2_ thin films were grown at 200°C and 230 mTorr using TiCl_4_ and H_2_O as the precursor vapors. During the growth process, the TiCl_4_ and H_2_O were delivered to the chamber using an N_2_ carrier gas at a flow rate of 50 sccm. The pulse time, purge time, and deposition rate were 250 ms, 1 s, and 0.05 nm/cycle, respectively. Moreover, top and cross-sectional views of the original and TiO_2_-coated nanoporous AAO thin films were observed via FE-SEM (Hitachi S-4800, Hitachi, Ltd., Chiyoda, Tokyo, Japan). The crystal structure was investigated via XRD (D/MAX-2500 V/PC, Tokyo, Japan) using Cu Kα radiation (*λ* = 0.154 nm) and a sampling width of 0.02° with a scan speed of 4°/min. Finally, HR-TEM (JEM 2100 F, JEOL Ltd., Akishima, Tokyo, Japan) was used to confirm the crystal structure of the as-deposited TiO_2_ thin films and filling process. The aluminum substrate was then completely removed using a saturated CuSO_4_ solution with a small amount of HCl (38 wt%), and the AAO template was etched in 6 wt% H_3_PO_4_ for 20 h at 23°C as shown in Figure 1a. To avoid any surface tension that may destroy the nanostructures, all samples were dried at room temperature for 12 h without annealing.

## Results and discussion

Figure 1a shows the schematic process for fabricating the free-standing TiO_2_ nanowire arrays. A 2 cm × 2 cm AAO template was prepared via the two-step anodization shown in Figure 1b. The samples fabricated by filling the AAO templates with TiO_2_ via sputtering or ALD are shown in Figure 1c. The surface color for the ALD-coated AAO template changed, while the sputtered AAO template remained unchanged. This macroscopic phenomenon primarily resulted from the multilayer interference effect [21], which also indicated that the ALD-coated TiO_2_ yielded better filling than the sputtering process. Finally, TiO_2_ nanowire arrays were fabricated via ALD by separately removing the aluminum substrate and AAO template via wet etching. In addition, the surface morphology of the fabricated nanowire arrays were characterized by SEM as shown in Figure 1d, which indicates highly ordered, large-scale, and free-standing TiO_2_ nanowire arrays.

**Figure 2:**
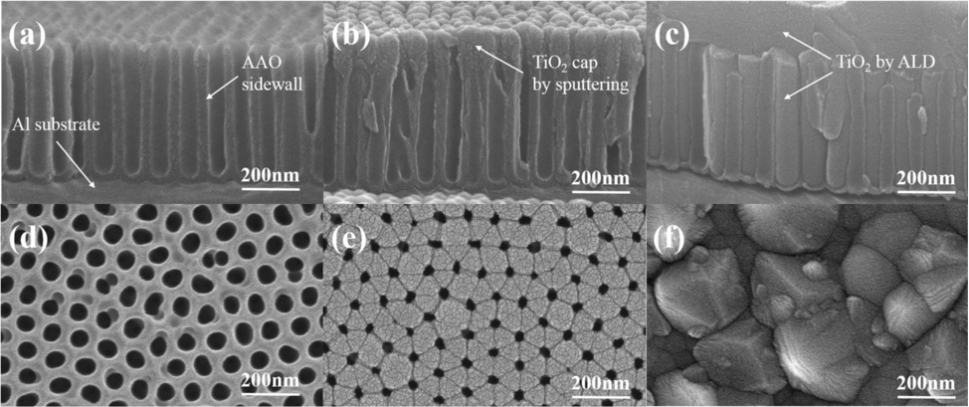
**FE-SEM images of 500-nm-thick nanoporous AAO thin film on the aluminum substrate. (a)** Cross-sectional results before and after TiO_2_ filling by **(b)** sputtering and **(c)** ALD, and **(d)** the surface results before and after TiO_2_ filling by **(e)** sputtering and **(f)** ALD.

Figure 2a,d shows the cross-sectional and top SEM images for a 500-nm-thick nanoporous AAO thin film with a 67-nm pore diameter and approximately 10^10^ cm^−2^ pore density. Figure 2a shows well-ordered cylindrical pores with a high aspect ratio which were observed after the two-step anodization. All of the pores run straight from top to bottom, which confirms that the nanoporous AAO template can be used to pattern both nanotubes and nanowires. In addition, the oxalic acid electrolyte dissolved parts of the AAO surface to form small, terminated pores [22], which appear as twinned pores (Figure 1d). Despite research into the cause of twinned pores being unclear, such a nanostructure would convert into an ideal hexagon with increased anodization time [23,24]. Figure 2b,e shows mushroom-like TiO_2_ thin films coated by sputtering onto AAO membranes. Due to the self-limited growth mechanism and high aspect ratio (7.5:1), most of the TiO_2_ films were concentrated on the surface of the AAO film surface. Increasing the deposition time decreased the nanopore diameter until they ultimately closed. However, the templates are well filled via ALD as shown in Figure 2c. Clear crystal grain boundaries were observed in Figure 2f, and there were no pinholes in the template surfaces.

**Figure 3:**
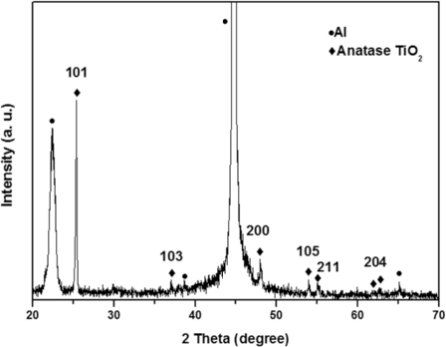
**XRD profile for the AAO template on Al substrate and TiO**_**2**_ **coated via the ALD process.**

To examine the TiO_2_ and AAO template interface and TiO_2_ thin film crystallinity, TiO_2_ nanowire arrays grown on the AAO membrane via ALD were analyzed by XRD and HR-TEM. The XRD profile for the AAO-assisted TiO_2_ synthesis is shown in Figure 3. A narrow, strong reflection peak from the (101) plane was identified at 2*θ* = 25.4°. Reflection peaks for the (103), (200), (105), (211), and (204) planes were also observed; however, their intensities were significantly weaker than the main (101) plane peak, which indicates that the ALD-coated TiO_2_ nanowires contained a pure anatase phase (JCP-DS No. 21–1272) with a mostly (101) orientation. The Al substrate peaks appeared in Figure 3 at 22.36°, 38°, 64°, 44.78°, and 65.18°; however, the AAO membrane peaks were absent. This finding may result from the AAO thin films not being annealed (higher than 200°C) during processing; the Al_2_O_3_ should be amorphous after the two-step anodization.

**Figure 4:**
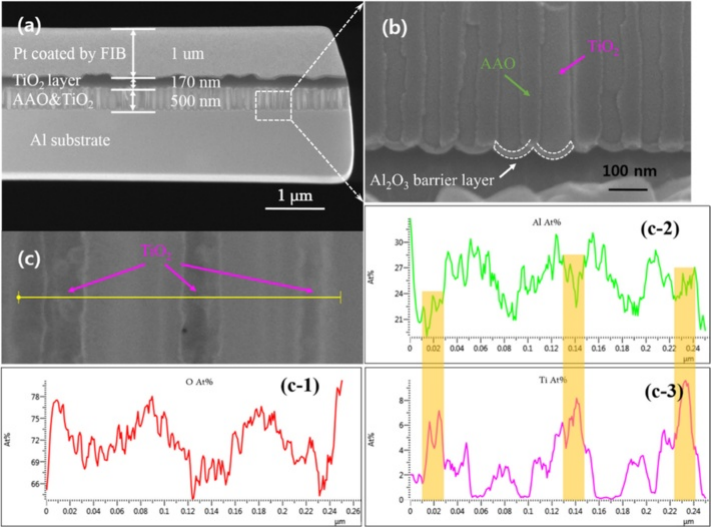
**FIB and TEM images for AAO template coated with TiO**_**2**_ **using ALD. (a)** Cross-sectional structure for the TEM samples. **(b)** The enlarged picture shows the barrier layer between the AAO template and Al substrate. **(c)** A cross section of the AAO and TiO_2_ arrays and EDX spectroscopic image for all elements for **(c-1)** O, **(c-2)** Al, and **(c-3)** Ti corresponding to the yellow line in **(c)**.

The TEM sample was prepared using a focused ion beam (FIB) as shown in Figure 4a. Because of the aluminum anodization mechanism [25,26], a viable 21.5-nm-thick barrier layer was created, as marked in Figure 4b. Accordingly, U-shaped TiO_2_ nanowires were observed after the ALD process. The TiO_2_ and Al_2_O_3_ nanowire arrays were confirmed via energy-dispersive X-ray spectrometer (EDS) mapping, which illuminated Ti and Al distribution. Figure 4c marks the EDS detection area with the yellow line, and all of the element distribution profiles are given in Figure 4c-1,c-2,c-3, which proved that both the Al and Ti concentrations periodically changed along the horizontal axis. In Figure 4c-2,c-3, the change in Al precisely exhibited an opposite tendency to the Ti across the entirety of the AAO arrays and TiO_2_ nanowires. The AAO nanopores were fully filled by the ALD process based on the cross-sectional structure and EDX spectroscopic image.

**Figure 5:**
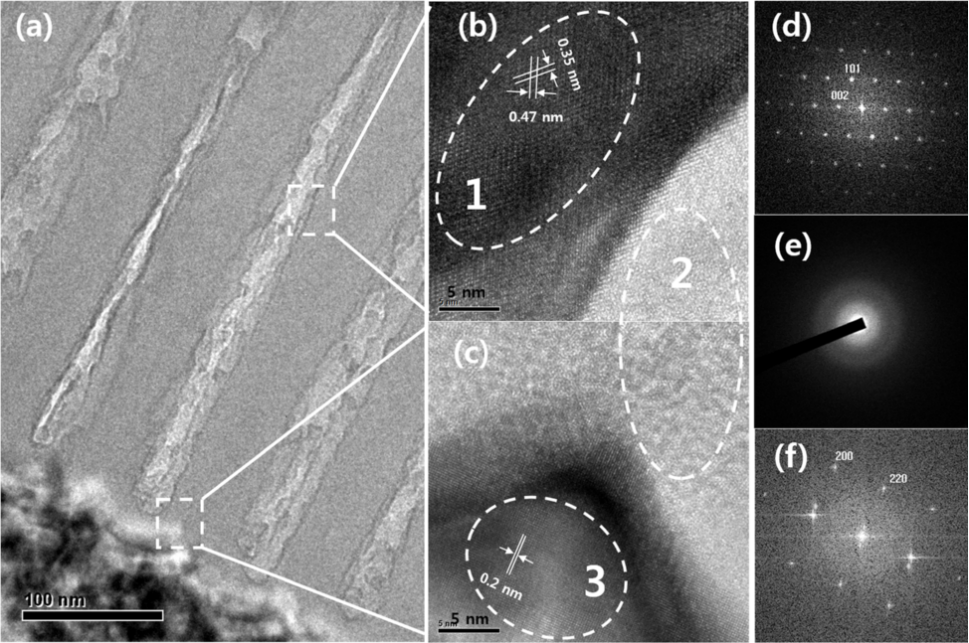
**TEM images of the AAO template coated with TiO**_**2**_ **using ALD. (a)** Cross-section of the TEM samples. **(b**, **c)** HR-TEM images of the anatase TiO_2_ (region 1), AAO (region 2), and Al substrate (region 3). **(d-f)** The FFT patterns for regions 1, 2, and 3, respectively.

To verify the above findings, TEM images of the AAO arrays and TiO_2_ nanowires were collected and are shown in Figure 5. Both the U-shaped TiO_2_ nanowire ends and bottom barrier layer were observed in the low-magnification TEM images (Figure 5a). The HR-TEM images of the TiO_2_ and Al_2_O_3_ interface and barrier layer were used to examine the crystal facets. The HR-TEM image in Figure 5b directly shows single-crystal anatase TiO_2_. Two confirmed lattice fringes were present with a distance of 0.35 and 0.47 nm, which corresponded to the {101} and {002} facets shown in the fast Fourier transformation (FFT) patterns (Figure 5d) in region 1 [27], respectively. Figure 5c shows the HR-TEM image for the AAO barrier layer and Al substrate. A 0.2-nm lattice fringe appeared in region 3, which corresponded to the {200} Al crystalline plane in the FFT patterns (Figure 5f) of region 3. However, crystalline Al_2_O_3_ was found in neither the HR-TEM images nor the FFT patterns as shown in region 2 and Figure 5e, respectively. These findings all agree with the XRD results.

**Figure 6:**
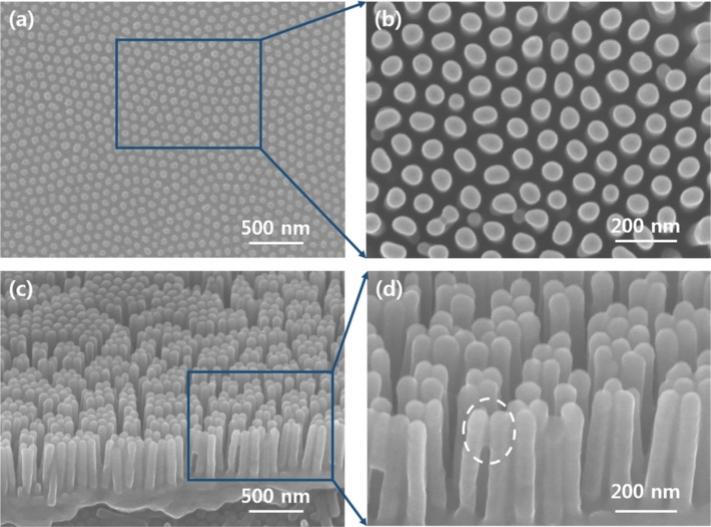
**SEM images of the transferred TiO**_**2**_ **nanowires.** The **(a**, **b)** top and **(c, d)** cross-sectional views of 400-nm-high free-standing TiO_2_ nanowire arrays with a diameter of 66.8 nm.

Finally, 400-nm-tall highly crystalline anatase TiO_2_ free-standing cylindrical nanowire arrays 67 nm in diameter formed using the AAO template via ALD as shown in Figure 6. The non-edge parts of the nanowire arrays were vertically arranged highly ordered TiO_2_ nanowires with uniform diameters. Only a few small nanowires are shown in the enlarged SEM image (Figure 6b) due to the twinned pores observed in Figure 2d. However, the cross-sectional images from the sample edge (Figure 6c,d) contain some anomalous nanowires. Notably, the two nanowires marked by the white circle in Figure 6d were amalgamate with each other at the top, which may be due to damage during the drying and transfer processes.

## Conclusions

In this study, self-ordered nanoporous AAO thin films with high aspect ratios and smooth sidewalls were successfully prepared via the two-step anodization of aluminum foil. The AAO thin film template was coated with a TiO_2_ layer via sputtering and ALD. The sputtered TiO_2_ layer formed a mushroom-like structure on the AAO template surface, which may form pinholes within the AAO nanopores. In contrast, the nanoporous AAO thin films were satisfactorily filled with TiO_2_ via the ALD approach. Furthermore, the ALD process promoted the crystallization of the as-deposited TiO_2_ films to form a single anatase TiO_2_ crystal with a low index of {101} and {002}. Based on these findings, combining the ALD approach with a wet etching process yielded highly ordered, large-scale, and free-standing TiO_2_ nanowire arrays with potential applications to be explored in further work, such as sensing, catalysis, and energy applications.

## References

[1] Kemell M, Pore V, Tupala J, Ritala M, Leskelä M (2007). Atomic layer deposition of nanostructured TiO_2_ photocatalysts via template approach. Chem Mater.

[2] Hernández S, Cauda V, Hidalgo D, Rivera VF, Manfredi D, Chiodoni A (2014). Fast and low-cost synthesis of 1D ZnO–TiO_2_ core–shell nanoarrays: characterization and enhanced photo-electrochemical performance for water splitting. J Alloy Compd.

[3] Li Y, Zhang LL, Wu WJ, Dai P, Yu XX, Wu MZ (2014). Hydrothermal growth of TiO_2_ nanowire membranes sensitized with CdS quantum dots for the enhancement of photocatalytic performance. Nanoscale Res Lett.

[4] Miller DR, Akbar SA, Morris PA (2014). Nanoscale metal oxide-based heterojunctions for gas sensing: a review. Sensor Actuat B.

[5] Yang HY, Cheng XL, Zhang XF, Zheng ZK, Tang XF, Xu YM (2014). A novel sensor for fast detection of triethylamine based on rutile TiO_2_ nanorod arrays. Sensor Actuat B.

[6] Liao JY, Manthiram A (2014). Mesoporous TiO_2_-Sn/C core-shell nanowire arrays as high-performance 3D anodes for Li-ion batteries. Adv Energy Mater.

[7] Thimsen EJ. Metal oxide semiconductors for solar energy harvesting. Electronic Theses and Dissertations. Washington University in St. Louis, Missouri. 2009:347.

[8] Wang CZ, Chen Z, Jin HB, Cao CB, Li JB, Mi ZT (2014). Enhancing visible-light photoelectrochemical water splitting through transition-metal doped TiO_2_ nanorod arrays. J Mater Chem A.

[9] Liu J, Yu XL, Liu QY, Liu RJ, Shang XK, Zhang SS (2014). Surface-phase junctions of branched TiO_2_ nanorod arrays for efficient photoelectrochemical water splitting. Appl Catal B.

[10] Gámez F, Plaza-Reyes A, Hurtado P, Guillén E, Anta JA, Martínez-Haya A (2010). Nanoparticle TiO_2_ films prepared by pulsed laser deposition: laser desorption and cationization of model adsorbates. J Phys Chem C.

[11] Mor GK, Varghese OK, Paulose M, Grimes CA (2010). Transparent highly ordered TiO_2_ nanotube arrays via anodization of titanium thin films. Adv Funct Mater.

[12] Tan BH, Zhang Y, Long MC (2014). Large-scale preparation of nanoporous TiO_2_ film on titanium substrate with improved photoelectrochemical performance. Nanoscale Res Lett.

[13] Ku SJ, Jo GC, Bak CH, Kim SM, Shin YR, Kim KH (2013). Highly ordered freestanding titanium oxide nanotube arrays using Si-containing block copolymer lithography and atomic layer deposition. Nanotechnology.

[14] Liu ZC, Liu ZF, Cui T, Dong LX, Zhang J, Han L (2014). Photocatalyst from one-dimensional TiO_2_ nanowires/synthetic zeolite composites. Mater Express.

[15] Ding GQ, Zheng MJ, Xu WL, Shen WZ (2005). Fabrication of controllable free-standing ultrathin porous alumina membranes. Nanotechnology.

[16] Masuda H, Fukuda K (1995). Ordered metal nanohole arrays made by a two-step replication of honeycomb structures of anodic alumina. Science.

[17] Liu SM, Gan LM, Liu LH, Zhang WD, Zeng HC (2002). Synthesis of single-crystalline TiO_2_ nanotubes. Chem Mater.

[18] Xie YR, Wei L, Wei GD, Li QH, Wang D, Chen YX (2013). A self-powered UV photodetector based on TiO_2_ nanorod arrays. Nanoscale Res Lett.

[19] Comstock DJ, Christensen ST, Steven T, Elam JW, Pellin MJ, Hersam MC (2010). Tuning the composition and nanostructure of Pt/Ir films via anodized aluminum oxide templated atomic layer deposition. Adv Funct Mater.

[20] Jeon G, Moon JS, Lee S, Lee JH, An BS, Hwang DY (2014). Individually aligned tubular ZnO nanostructures on solid substrates. Mater Lett.

[21] Yang CY, Shen WD, Zhang YG, Ye ZJ, Zhang X, Li K (2014). Color-tuning method by filling porous alumina membrane using atomic layer deposition based on metal-dielectric-metal structure. Appl Optics.

[22] Mozalev AS, Magaino HI (2001). The formation of nanoporous membranes from anodically oxidized aluminium and their application to Li rechargeable batteries. Electrochim Acta.

[23] Li FY, Zhang L, Metzger RM (1998). On the growth of highly ordered pores in anodized aluminum oxide. Chem Mater.

[24] Masuda H, Yada K, Osaka A (1998). Self-ordering of cell configuration of anodic porous alumina with large-size pores in phosphoric acid solution. Jpn J Appl Phys.

[25] Tian ML, Xu SY, Wang JG, Kumar N, Wertz E, Li Q (2005). Penetrating the oxide barrier in situ and separating freestanding porous anodic alumina films in one step. Nano Lett.

[26] Liang YC, Wang CC, Kei CC, Hsueh YC, Cho WH, Perng TP (2011). Photocatalysis of Ag-loaded TiO_2_ nanotube arrays formed by atomic layer deposition. J Phys Chem C.

[27] Pan J, Liu G, Lu GQ, Cheng HM (2011). On the true photoreactivity order of {001}, {010}, and {101} facets of anatase TiO_2_ crystals. Angew Chem.

